# Feasibility of Stereotactic Body Radiation Therapy on Unresectable Stage III NSCLC with Peripheral Primary Tumor: A Prospective Study (GFPC 01-14)

**DOI:** 10.3390/curroncol28050324

**Published:** 2021-09-28

**Authors:** Isabelle Martel-Lafay, Isabelle Monnet, Audrey Lardy-Cleaud, Serge Danhier, Naji Salem, Olivier Gallocher, Pierre Fournel, Christos Chouaid, Olivier Bylicki, the GFPC 01-14 Investigators

**Affiliations:** 1 Centre Léon Bérard, Radiotherapy, 69008 Lyon, France; isabelle.martel-lafay@lyon.unicancer.fr; 2 CHI Créteil, 94010 Créteil, France; isabelle.monnet@chicreteil.fr; 3 Centre Léon Bérard, Direction de la Recherche Clinique et de l′Innovation, 69008 Lyon, France; audrey.lardy-cleaud@lyon.unicancer.fr; 4 Centre François Baclesse, 14000 Caen, France; serge.danhier@ch-cornouaille.fr; 5 Institute Paoli Calmettes, 13009 Marseille, France; salemn@ipc.unicancer.fr; 6 Clinique Pasteur—Atrium, 31300 Toulouse, France; ogallocher@clinique-pasteur.com; 7 Institut de Cancérologie, 42270 Saint-Etienne, France; pierre.fournel@icloire.fr; 8 Inserm U955, UPEC, IMRB, équipe CEpiA, Créteil, CHI Créteil, 94010 Créteil, France; 9 HIA Toulon, 83800 Toulon, France; olivier.bylicki@intradef.gouv.fr

**Keywords:** stereotactic body radiation therapy, concomitant radiochemotherapy, unresectable stage III NSCLC

## Abstract

Concomitant radiochemotherapy (RTCT) is the standard treatment for unresectable stage III non-small cell lung cancer (NSCLC). However, in patients with a peripheral primary tumor, the irradiated volume may include a large portion of normal lung and RT-CT is not possible. This multicenter phase II trial in unresectable stage III NSCLC with peripheral primary tumor evaluated the feasibility of stereotactic body radiation therapy (SBRT) in peripheral tumor after concomitant radio-chemotherapy (RT-CT). Nineteen patients were included and analyzed (median age, 60.9 years; male, 78%; adenocarcinoma, 74%; median size of peripheral primary tumor, 19 mm). At 6 months, the disease control rate was 79% (15/19). SBRT toxicity was generally mild with one (5%) patient having grade 3 lung toxicity. Recruitment for this study was stopped prior to completion, firstly due to the approval of adjuvant durvalumab after RT-CT, which was not anticipated in the design, and secondly due to the small number of stage III NSCLC patients with a peripheral tumor that was accessible to SBRT. Nevertheless, the combination of RT-CT and SBRT appeared to be feasible and safe.

## 1. Introduction

Lung cancer is the most common cancer worldwide and is responsible for 2 million deaths per year [1]. Non-small cell lung cancer (NSCLC) accounts for more than 80% of primary bronchial cancers and, at the time of diagnosis, 30% of patients have locally advanced disease (stage III) [2,3].

Concomitant radio-chemotherapy (RT-CT) is the treatment recommended by European guidelines for unresectable stage III NSCLC [4]. One of the chemotherapies with the best benefit/risk ratio is the cisplatin–vinorelbine combination [5]. The possibility of using oral vinorelbine (Navelbine^®^) at a dose of 40 mg/m^2^ at D1 and D8 coupled with cisplatin (80 mg/m^2^ at D1) for each cure during conformational radiotherapy was demonstrated in the VINCR trial [6]. However, locoregional control is generally insufficient. In addition, the limiting toxicity of radiotherapy, particularly lung toxicity, does not allow optimal treatment of large tumor volumes, which is the case in patients with mediastinal lymph node invasion and primary peripheral tumor [7].

Stereotactic body radiation therapy (SBRT) is a technique for delivering high doses to a limited volume. Its advantages are very moderate lung toxicity due to the small volume of lung irradiated and a better efficiency due to the high doses delivered per session in a highly accurate way [8,9].

In this study on unresectable stage III NSCLC with peripheral primary tumor, we prospectively evaluated the feasibility of adding SBRT to the primary tumor after RT-CT for hilar or mediastinal nodal involvement.

## 2. Materials and Methods

### 2.1. Type of Study and Design

This was a single-stage multicenter national open-label phase II trial in patients with unresectable stage III NSCLC and peripheral primary tumor. The primary objective was to evaluate the feasibility of SBRT in peripheral lung tumor after RT-CT for unresectable stage III NSCLC. A peripheral tumor was defined as a tumor located 2 cm or more from the mediastinal organs, as shown in Timmerman′s diagram [10].

### 2.2. Patients

**Figure 1:**
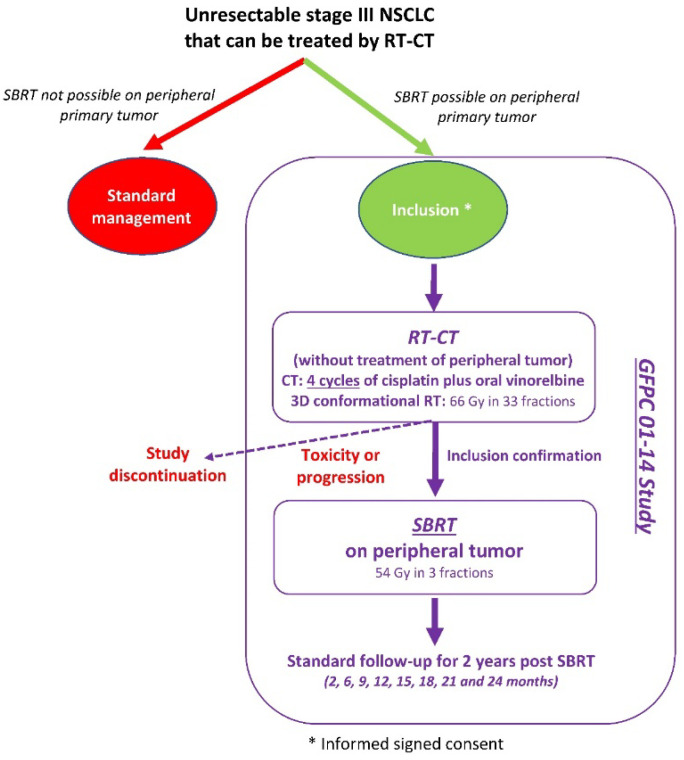
Study design.

Patients were included after completion of RT-CT (Figure 1). The main inclusion criteria were: age ≥ 18 years; ECOG (Eastern Cooperative Oncology Group) performance status (PS) 0–1; histologically proven NSCLC; unresectable stage III, i.e., T1 or T2 or T3 ≤ 5 cm and N2 or N3 (only contralateral mediastinum or homolateral supraclavicular); peripheral primary tumor ≥1 cm and ≤5 cm; patients eligible for RT-CT; adequate biological parameters; forced expiratory volume (FEV) ≥1 L or ≥30% of the theoretical value. The main exclusion criteria were: small cell or large cell neuroendocrine carcinoma, metastatic disease, resectable tumor.

After RT-CT, inclusion was confirmed for patients who received at least 3 cycles of CT and at least 60 Gy during RT, without lung or esophageal toxicity ≥grade 3, with no progression of peripheral primary tumor (≤5 cm).

### 2.3. Treatments

RT-CT included one induction cycle with cisplatin 80 mg/m^2^ at D1 and oral vinorelbine (Navelbine^®^) 60 mg/m^2^ at D1 and D8, followed by cisplatin 80 mg/m^2^ at D22, D43 and D64 during irradiation and oral vinorelbine 40 mg/m^2^ at D22, D29, D43, D50, D64, and D71. RT delivered 66 Gy from D22 in 33 fractions (2 Gy per session; 5 sessions per week) on mediastinal and hilar lymph node involvement without treating the peripheral tumor.

SBRT for primary peripheral tumor started within 3–4 weeks after the end of RT-CT and delivered 54 Gy in 3 fractions at D1, D3, and D5. SBRT was delivered either with a dedicated accelerator (Cyberknife^®^, Vero^®^) or a LINAC (VersaHD^®^ Elekta, TrueBeam^®^ Varian, Stockholm, Sweden. Patients treated with Cyberknife^®^ and Vero^®^ benefited from tumor tracking, which allowed to reduce the dose to healthy lungs and normal tissues. For the LINAC treatment, multiple (10 to 12) coplanar conformational small fields or VMAT^®^ (Volumetric Modulated Arc Therapy) were used. For tumor of the lower lobes, abdominal compression (Bodyframe^®^) was used for reducing the amplitude of respiratory movements. In case of pulmonary or esophageal toxicity of grade ≥3 or tumor progression (>5 cm) preventing timely completion of SBRT, the patient discontinued the study.

### 2.4. Data Collection and Evaluations

The following data were recorded: demographics, medical and surgical history, prior cancer treatment, and clinical examination including ECOG PS. Paraclinical assessments were performed before RT-CT: biological work-up, pulmonary function tests, electrocardiogram, bronchial fibroscopy, PET scan, brain imaging, computed tomography of chest, abdomen, and pelvis. A follow-up visit was performed at 2 months and then every 3 months up to 2 years post SBRT. Tumor response was assessed according to RECIST 1.1 criteria by CT scan and PET scan. Local control was defined as tumor control and locoregional control as tumor and mediastinal adenopathy control.

Safety was assessed according to National Cancer Institute Common Toxicity Criteria, version 4.0.

### 2.5. Statistical Analysis

The primary endpoint was the local control rate at 6 months. The number of subjects to be included was calculated using Fleming′s one-step Phase II design [11]. The following assumptions were made: *p*_0_ = 40% was the maximum value of local control rate at 6 months after SBRT, which did not justify further evaluation of the proposed therapeutic strategy; *p*_1_ = 55% was the minimum value, which justified further evaluation of the therapeutic strategy. With an alpha error set at 5% and a power of 80%, it was necessary to include 67 patients in the study to reject the null hypothesis H_0_, *p* ≤ *p*_0_, versus the alternative hypothesis H_1_, *p* ≥ *p*_1_, in a one-sided setting. In order to take into account patients not assessable for the primary endpoint (patients with metastatic progression before 6 months estimated at 5%), 70 patients had to be included.

## 3. Results

A total of 25 patients were enrolled from 22 December 2015 to 23 April 2018 in 12 centers. SBRT was not feasible in 6 patients after RT-CT (<3 chemotherapy cycles, *n* = 4; radiotherapy < 60 Gy, *n* = 4; lung or esophageal grade 3 toxicity, *n* = 4; missing data, *n* = 1; missing data for progression of peripheral primary tumor, *n* = 1).

Patients had a median age of 60.9 years and 78% were male (Table 1). Histology confirmed adenocarcinoma in the majority of cases (74%). The median size of the peripheral primary tumor was 19 (10 to 36) mm.

For RT-CT, patients received a median dose of 66 Gy in 33 fractions with oral vinorelbine plus cisplatin for 4 cycles (concomitantly for 3 cycles) (Table 1). In patients with confirmed inclusion after RT-CT, a median dose of 54 Gy on the 80% isodose (10 to 62 Gy) was administered to the peripheral tumor with SBRT in a median of 3 fractions (3 to 5) (Table 1).

At 6 months, the local response rate (RECIST 1.1) of peripheral primary tumor was partial response (PR) for 68% (13/19), stable disease (SD) for 11% (2/19), progressive disease (PD) for 16% (3/19); one patient died before 6 months. Objective response rate (CR + PR) was 68% (13/19), and disease control rate (CR + PR + SD) was 79% (15/19).

During SBRT, one patient had a grade 1 cough and grade 1 hemoptysis. After completion of SBRT, three patients experienced adverse events related to SBRT: grade 3 lung toxicity (*n* = 1), grade 2 bronchial stenosis (*n* = 1), and grade 1 cough (*n* = 1).

After a median follow-up of 58 (95% CI, 51.7–64.3) months, median OS was 51.6 (95% CI, 42.7–62.9) months. No patient had isolated recurrence in irradiated sites.

## 4. Discussion

Unresectable stage III NSCLC with peripheral primary tumor is a rare but complex management issue. In a significant number of cases, it is difficult to perform standard RT-CT treatment satisfactorily. In this prospective feasibility study, we evaluated the possibility of SBRT treatment of the peripheral tumor at the end of the RT-CT sequence. The feasibility rate was 76% (19/25) with control of the disease in 79% (15/19) of cases at 6 months and acceptable tolerance.

SBRT superseded conventional radiation therapy (CRT) for the treatment of patients with inoperable early-stage NSCLC over a decade ago. However, the direct comparisons of the outcomes of SBRT and CRT remain controversial. A phase 3, open-label, randomized controlled trial in patients with inoperable peripherally located stage 1 NSCLC (TROG 09.02 CHISEL trial) showed that compared with standard radiotherapy, SBRT resulted in superior local control of the primary disease without an increase in major toxicity [12]. More recently, a meta-analysis in stage 1 NSCLC including 17,973 patients (SBRT, *n* = 7395; CRT, *n* = 10,578) showed that SBRT was associated to a superior survival in terms of OS (HR 0.66, 95% CI 0.62–0.70, *p* < 0.00001) and PFS (HR 0.34, 95% CI 0.25–0.48, *p* < 0.00001) with a better tolerance; the SBRT group had a significantly lower rate of dyspnea, esophagitis, and radiation pneumonitis; no significant difference was found in grade 3–5 adverse events [13]. A steep dose–response relationship exists with high rates of durable local control when physical doses of 43–50 Gy are delivered in 3 to 5 fractions [14].

There are few data on the use of SBRT in stage III NSCLC, particularly in patients with peripheral tumor. The efficacy of SBRT has been more widely studied in localized forms of NSCLC. Thus, a recent meta-analysis showed that in inoperable early stage I NSCLC, SBRT was associated with better survival and lower rates of dyspnea, esophagitis, and radiation pneumonitis than conventional RT [13]. In stage III, the studies are more focused on the possibility of delivering a higher dose of radiotherapy locally. For example, a pilot study used SBRT after neoadjuvant chemotherapy and surgery in 10 patients with stage IIIA (multistation N2) or IIIB NSCLC (30 Gy SBRT to the primary lesion); the overall survival and progression-free survival rates at 2 years were 68% (90% CI 36–86) and 40% (90% CI 16–63) without local recurrence, respectively [15]. Pulmonary-related grade 3 adverse events were experienced by two patients. Likewise, a phase I study using RT-CT with SBRT boost for unresectable stage III NSCLC (primary tumor < 8 cm and N1 or N2 lymph nodes < 5 cm) in 15/19 (79%) of enrolled patients concluded that an SBRT boost dose of 10 Gy × 2 combined with 44 Gy of chest chemoradiation was safe without grade 3 or higher toxicities [16].

Radical-intent hypofractionated radiotherapy for locally advanced NSCLC remains a challenge. In a systematic review of the literature, Kaster et al. selected 33 studies (number of fractions, 15–35; dose per fraction, 2.3–3.5 Gy; delivered dose, 45.0–85.5 Gy) [17]. Fifteen of the studies included concurrent chemotherapy. OS was associated with tumor biological effective dose (Pearson correlation coefficient, 0.34–0.48). For both concurrent and nonconcurrent chemoradiotherapy, acute pulmonary, late esophageal, and late pulmonary incidences of toxicity ranged from 1.2% to 12.2%, but with 95% CI that included zero. Acute esophageal toxicity had the greatest incidence of all toxicities (14.9%, 95% CI 0.7–29.1). Therefore, there was a moderate linear relationship between biological effective dose and OS with concurrent chemotherapy and greater acute esophageal toxicity. The authors concluded that improving outcomes in stage III NSCLC may involve some form of hypofractionation in the context of systemic concurrent therapy. Studies of radiation dose escalation with modified fractionation schedules in this setting for locally advanced NSCLC were recently analyzed by Zehentmayr et al. [18]. In this analysis, dose escalation above the conventional 60 Gy using modified radiation fractionation schedules and shortened overall treatment time yielded similar OS and local regional control regardless of treatment sequence.

In these patients, immunotherapy has significantly changed the management. A recent update on the phase 3, placebo-controlled PACIFIC trial of patients with unresectable, stage III NSCLC without disease progression after concurrent chemoradiotherapy reported that consolidative durvalumab was associated with an estimated median OS of 47.5 vs. 29.1 months and estimated 4-year OS rates of 49.6% vs. 36.3% for durvalumab vs. placebo [19]. Several studies have established that radiotherapy in combination with immunotherapy has a strong synergistic effect [20]. Radiotherapy changes the tumor microenvironment, generates local inflammation reactions, and enhances immunostimulatory effects, which are able to improve immunotherapy efficacy. To date, three phase 3 trials have been published on radiotherapy and sequential immunotherapy with variable outcomes, ranging from no significant difference [21,22] to absolute differences in OS of 13.5% after 3 years [19]. No phase 3 randomized trials have been published on the simultaneous combination of radiotherapy with immuno-oncology. Several trials are presently ongoing using immunotherapy as induction therapy followed by chemoradiotherapy, or in addition to concurrent chemoradiotherapy. These studies will make it possible in the coming years to determine the ideal sequence for the management of locally advanced NSCLC. For the patients included in our study, this sequence could thus combine SBRT started just after the end of the concomitant chemoradiotherapy, followed by consolidation with durvalumab.

The main limitation of the study is the small sample size. Only 19 patients out of the 67 planned could be analyzed. First, a small number of stage III NSCLC patients presented with a peripheral tumor that was accessible to SBRT. Second, patient enrollment was stopped before completion due to the approval of adjuvant durvalumab after RT-CT, which was not anticipated in the design [23].

## 5. Conclusions

Despite the limitations of the study, the combination of RT-CT and SBRT appears to be feasible and safe in patients with unresectable stage III NSCLC and peripheral primary tumor.

## Figures and Tables

**Table 1:** Patient and treatment characteristics.

Patients and Treatment Characteristics	*n* = 19
Age, years, median (range)	60.9 (38–76)
Male gender, *n* (%)	15 (78)
Stage, *n* (%)	
IIIA	10 (53)
IIIB	9 (47)
Histology, *n* (%)	
Adenocarcinoma	14 (74)
Squamous-cell carcinoma	2 (10.5)
Undifferentiated carcinoma	2 (10.5)
Other	1 (5)
ECOG performance status, *n* (%)	
0	13 (68)
1	6 (32)
Tobacco status, *n* (%)	
Current smoker	8 (42)
Former smoker	11 (58)
Peripheral primary tumor size, mm, median (range)	19.0 (10–36)
Radiotherapy	
Number of fractions performed, median (range)	33 (30–33)
Total dose, Gy, median (range)	66 (0–66)
Chemotherapy	
Number of cycles, median (range)	4 (3–4)
SBRT	
Total volume GTV, mm, median (range)	3.0 (1–24)
Number of fractions performed, median (range)	3 (3–5)
Radiotherapy duration, days, median (range)	5 (3–16)
Total dose administered at prescription isodose, Gy, median (range)	54 (10–62)
Prescription isodose, %, median (range)	80 (70–99)
Type of radiotherapy, *n* (%)	
CyberKnife	9 (47)
Accelerator	2 (11)
Novalis	1 (5)
TrueBeam	1 (5)
Vero	2 (11)
Other	4 (21)

GTV, gross tumor volume; SBRT, stereotactic body radiation therapy.

## Data Availability

Individual participant data that underlie the reported results will not be made available.
